# Evidence for return to work following complex orthopaedic injury - a scoping review

**DOI:** 10.1177/10519815251334596

**Published:** 2025-04-21

**Authors:** Andreas Conte, Ellisiv Clarke, Aswinkumar Vasireddy

**Affiliations:** 1 Faculty of Life Sciences & Medicine, King's College London, London, UK; 2 Department of Trauma and Orthopaedics, King's College Hospital, London, UK

**Keywords:** biopsychosocial model, rehabilitation, prognosis, musculoskeletal injury, orthopaedic, trauma, prognosis

## Abstract

**Background:**

The rates of return to work (RTW) following complex orthopaedic injury are low. No review has summarised current knowledge and prognostic factors for RTW in these patients.

**Objectives:**

This scoping review presents the nature and the extent of current research evidence for the prognostic factors of RTW following complex orthopaedic injury.

**Methods:**

This review was designed using the PRISMA-ScR checklist. Online databases were searched for articles (1969–2023) which covered: return to work, complex orthopaedic injury, and prognostic factors. Complex orthopaedic injury is defined as multiple fractures, open fractures, high energy pelvic injuries or polytrauma with related orthopaedic injury. Data were extracted and placed into an evidence table.

**Results:**

Eleven studies were eligible for inclusion. All but one were prospective cohort in design with small patient numbers. There was a skew for studies of open tibial fractures. There was limited breadth of tested prognostic factors. The only commonly reported statistically significant prognostic factors were age, ISS classification and nature of work. Endpoints for RTW ranged from 3 months to 8 years, preventing the pooling of data for meta-analyses.

**Conclusion:**

In summary, there is a limited understanding of prognostic factors for RTW following complex orthopaedic injury. Estimates for RTW following these injuries range from 16–90%. The authors recommend that future studies take a holistic approach, using the biopsychosocial model, when investigating prognostic factors for RTW in these patients. This study offers guidance on qualitative investigation, investigative variables and mechanisms to achieve this.

## Introduction

Complex orthopaedic injury is defined as “multiple fractures, open fractures, high energy pelvic injuries and polytrauma with related orthopaedic injury”.^1^ These injuries effect 50% of Major Trauma patients,^2^ equating to 10,000 people per year within the UK.^3^ The immediate cost of treatment is estimated at £0.2 billion a year, but the costs of further hospital treatments, rehabilitation and home care support remain unknown.^3^ In total, it is estimated that complex orthopaedic injuries are responsible for £1.5–2 billion in annual lost economic output.^3^

Following these injuries, patients are treated at specialist hospitals within the national Major Trauma Network^4^ which have the personnel and equipment to treat and rehabilitate the most critically injured patients. As part of their recovery, NICE national guidelines^5^ emphasise that these patients should receive a comprehensive rehabilitation programme, addressing their physical, cognitive and psychological recovery. This includes multi-disciplinary rehabilitation within hospital and following discharge, aiming to address the biopsychosocial^6^ domains of their recovery.^7,8^ However, the limited academic analysis suggests that these guidelines are applied inconsistently,^9^ contributing to long-term hospital treatment and greater hospital costs.^10^

The academic literature consistently finds work disability to be associated with orthopaedic injury,^11–13^ leading to personal and societal strain. If a patient fails to return to work (RTW), they likely suffer from worse health, psychological and social outcomes, including more frequent hospital visits, increased depressive symptoms, and financial pressures.^14^ These pressures can also extend to the patient's family and caregivers, causing employment and financial burdens. Importantly, however, the same literature shows that when a patient is able to RTW, their psychological well-being, self-esteem, and social connectedness increase, whilst the societal burden decreases.^15,16^ Therefore, RTW is a critical outcome for patients, their family, and wider society.

The biopsychosocial model^6^ can be used to highlight the challenges specific to RTW in complex orthopaedic patients. This may include newly acquired physical limitations, psychological trauma impacting mood, or social barriers related to workplace modifications.

Rates of RTW vary significantly owing to the heterogeneous nature of orthopaedic injury.^11,13^ Even amongst complex orthopaedic injuries, epidemiological studies report varying rates of RTW between 20%^17^ and 80%.^18^ Despite the considerable societal impact, there have only been two reviews exploring the prognostic factors for RTW following orthopaedic injury.^11,13^ These reviews identified injury severity, age, pain, self-efficacy, education level, type of work and compensation status as prognostic factors. However, these papers also pooled together orthopaedic injuries ranging from isolated to global, and minor to severe, generalising their results. Indeed, patients with complex orthopaedic injury would be expected to have more pronounced or even different prognostic factors compared to orthopaedic injuries more generally. To date, no review has explored RTW following complex orthopaedic injury.

Given the very limited academic study into prognostic factors for RTW in complex orthopaedic injuries, a scoping review has been chosen as the most appropriate method of exploring this topic.^19^ This paper aims to explore the variety of the literature, map current evidence, and identify any gaps in knowledge.^20^ This information could then be used to inform new areas for research, which can contribute to improved outcomes for patients with complex orthopaedic injury.

## Methods

This review was designed using published guidance for scoping reviews, which included a PRISMA-ScR checklist.^19^

### Literature search and eligible studies

A literature search of studies in English was conducted between January 1969 and December 2023 on PubMed, MEDLINE and EMBASE databases. The literature search was performed in April 2024. Articles had to cover three areas: RTW, complex orthopaedic injury and prognostic factors. The search terms were: “return to work” OR “work absence” OR “work disability” OR “time off work” AND “orthopaedic trauma” OR “orthopaedic injury” OR “fractures” OR “polytrauma” OR “multiple fractures” OR “open fracture” OR “high energy pelvic trauma” AND “prognosis” OR “prognostic factors” OR “risk factors” OR “outcome”.

The literature search was conducted by one investigator. References were then uploaded to Rayyan,^21^ where duplicates were removed. Two investigators made the first blinded selection of articles based on the title and abstracts. The selections were then unblinded, and full text articles reviewed. The final decision for article inclusion was based on consensus.

**Figure 1.**
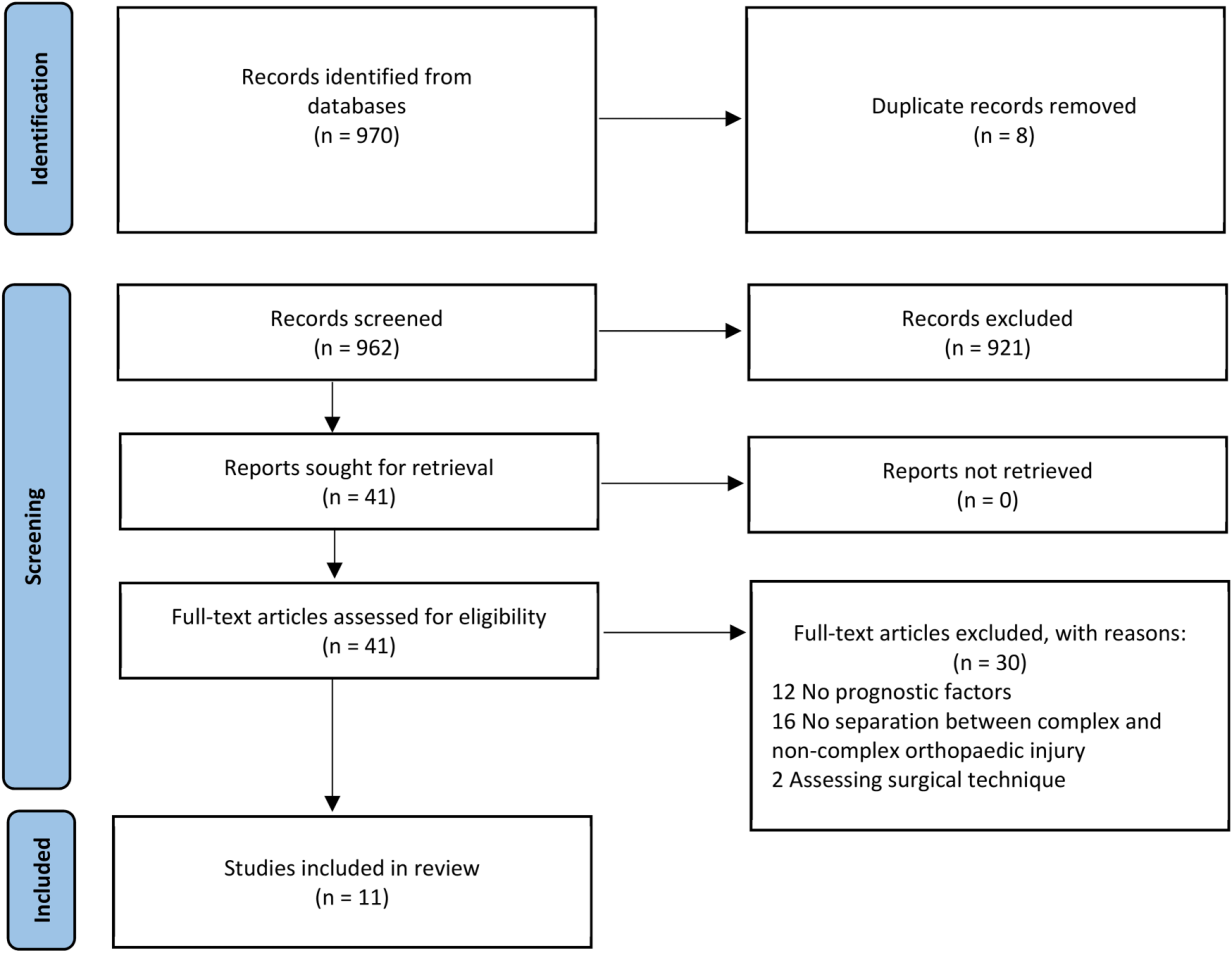
PRISMA flow diagram, showing the search strategy and number of included and excluded studies.

Reviews, opinion papers, editorials, conference abstracts and case reports were not eligible for inclusion. Studies which included patients with major thoracic, abdominal, and brain injuries were also excluded, to reduce the risk of confounding factors. The search strategy is shown in Figure 1.

### Data extraction

Data were extracted from selected studies into an evidence table, summarising the author, injury type, number of patients, follow up period, statistically significant predictors for RTW, statistically insignificant predictors for RTW, and rate of RTW at final follow up. A formal quality assessment of eligible studies was not performed as this is beyond the remit of a scoping review. The data collected in the evidence table was used to define the main themes of the research.

## Results

In summary, this scoping review found a limited size of literature looking into prognostic factors for RTW following complex orthopaedic injury. There is no meta-analysis, systematic review or randomised controlled trials on this topic. Strikingly, there was almost a complete lack of studies conducted in developing nations. Regarding types of injury, there was a skew towards open fractures of the tibia.

### Searches

The search retrieved 970 articles. After the removal of duplicates, 962 underwent title and abstract screening. Of those, 41 underwent full text screening, with 11 meeting the inclusion criteria of RTW, complex orthopaedic injury and prognostic factors. These studies are summarised in Table 1.

**Table 1.** Characteristics of included studies.

	Author	Country	Injury	Patients	Follow up	Statistically significant predictors for RTW	Statistically insignificant predictors for RTW	Return to work (%) at endpoint
Open Fracture	Belangero et al., 2022^23^	Brazil	Tibial	57	3 months	Gait status Age Infection SF-12 physical component score SF-12 mental component score Fracture status on radiograph (healed vs not healed)	Mechanism of injury AO classification Gustilo et al., classification ASA classification Time to initial surgery Time to antibiotic initiation Time to definitive surgery Soft tissue management Initial bone management Time to definitive surgery	70.6%
	Fioravanti et al., 2018^24^	France	Tibial	24 amputation 27 conservative	Up to 5 years		Amputation versus conservative treatment	60% amputation 47% conservative
	Frisvoll et al., 2018^25^	Norway	Tibial	14 amputation 22 reconstruction	Up to 8 years		Amputation versus conservative treatment	73% reconstruction 50% amputation
	Levy et al., 2022^26^	United States	Tibial	857	1 Year	> 40 years of age Physically demanding job Most severe injuries		47.2%
	METRC., 2021^27^	United States	Severe distal tibial, ankle, mid/hindfoot	639	1.5 years		Amputation versus conservative treatment	Not reported
	Sethuraman et al., 2021^28^	India	Tibial	78	3 years	Amputation versus conservative treatment		Not reported
High Energy Pelvic Trauma	Aprato et al., 2017^29^	Italy	Acetabular fracture	108	Up to 6 years	Time to definitive surgery ICU admission Sedentary work	Gender Age Fracture complexity Sedentary work Job sector ASA classification	90%
	Gabbe et al., 2015^30^	Australia	Pelvic ring fracture	111	2 years	ISS classification	Bladder Injury Pelvic fracture management	59%
Multiple Fractures	Smith et al., 2005^31^	United States	Bilateral lower limb	Unilateral salvage/amputation = 8 Bilateral salvage = 14 Bilateral amputation = 10	2 years	Bilateral vs unilateral involvement		66.7% unilateral salvage/amputation 21.4% bilateral salvage 16.0% bilateral amputation
Polytrauma	Kuhlman et al., 2014^32^	Denmark	Trauma severity	1722	6.2 years (median)	ISS classification (1–15 vs 16–75)		
	Larsen et al., 2016^33^	Denmark	Polytrauma	53	1 year		Quality of life (Eq5d-5Ln score)	32%

### Study design

Ten of the eleven articles followed an observational cohort study design. The other study was a case-control. Nine of the studies were prospective, one was a mix of retrospective and prospective, one was retrospective.

### Sample size

Six studies had less than 100 subjects, and one had over 1000. Patients lost to follow-up ranged from 0–30%.

### Author affiliation

The most common first author affiliation was the United States, in keeping with American prevalence in academic publishing.^22^ The next most common author affiliation was Denmark, with two studies identified. Brazil, France, Norway, India, Italy, and Australia led one study each.

### Type of injury

Six articles studied open fractures, five of which focused exclusively on the tibia. The other study included the ankle and midfoot. Two studies focused on high energy pelvic trauma, one focused on pelvic ring fractures, the other on acetabulum fractures. One study focused on multiple fractures involving both lower limbs. Two studies focused on polytrauma, one orthopaedic polytrauma, the other the extent of injury severity.

### Year of publication

The year of publication ranged from 2005 to 2022, with a skew towards later years.

## Discussion

### Type of complex orthopaedic injury

The majority of the presented literature is focused on open fractures of the tibia. There are no studies of prognostic factors for RTW of other open long bone fractures. This may be because the tibia is the most common open long bone fracture, involved in 51.3% of all open fractures, compared to the radius/ulnar at 26.2%, femur at 7.4%, and humerus at 5.4%.^34^ With an estimated prevalence of 30.7/10^5^/year, open fractures are common injuries which cause significant work disability.^35^ Therefore, further research is needed to better explore and describe the predictive factors for RTW in these injuries.

Only two studies looked at prognostic factors for RTW in high energy pelvic trauma. One focused on the acetabulum,^29^ the other on the pelvic ring.^36^ Neither of these studies assessed the same prognostic factors for RTW, and with small patient populations, struggled to reach statistically significant conclusions. Only one study looked at multiple fractures, however, the only predictive factor tested was bilateral versus unilateral involvement, and they did not present any statistical analysis.^31^ Two studies looked at polytrauma patients, but only tested two prognostic factors – ISS classification^32^ and a quality-of-life assessment.^33^ These studies highlight the lack of thorough research into complex orthopaedic injuries and their implications for RTW.

### Time of follow up

There is no common timepoint when reporting rates of RTW. Some studies report the rates of RTW at final follow up, creating significant heterogeneity within the same study.^25,32^ Other studies use a fixed endpoint, ranging from three months to three years post injury. This makes it challenging to compare prognostic factors for RTW between studies and over time.

To mitigate this effect, Duong et al.,^11^ divided predictors into early phase (<6 months) and late phase (>6 months) when reviewing RTW in all orthopaedic injuries. They found different predictors for RTW based on time, further explored in *4.3 Prognostic Factors* below. In response, the authors of this paper recommend that future research into this topic analyses predictors at <6 months and >6 months from date of injury to enable future reviews and meta-analyses.

### Variety of orthopaedic injury

There are several epidemiological studies exploring different types of complex orthopaedic injury which use RTW as a dependent variable.^17,37,38^ However, these studies do not attempt to explore predictors linked to RTW. Those that do often pool complex orthopaedic injuries with less severe injuries,^39,40^ generalising the findings of prognostic factors.^11,13^ To combat this, specific research into complex orthopaedic injuries is required, which would benefit from a holistic approach using the biopsychosocial model.^6^

### Prognostic factors – biopsychosocial model

#### Biological factors

At <6 months, Duong et al.,^11^ only identified injury severity as a strong predictor for RTW. Similarly, this review describes the SF-12 physical component score as a strong predictor of RTW at 3 months.^23^ Beyond 6 months, Duong et al.,^11^ describe other biological factors, in addition to injury severity, such as age and level of pain as strong predictors of RTW. Similarly, this review reports injury severity^26,30,32^ and age^26^ to be commonly reported predictors of RTW in studies longer than one year.

However, other biological factors not found to be significant by Duong et al.,^11^ were found to be predictive in articles included in this review. This includes ICU admission^29^ and time to definitive surgery.^29^ These examples may suggest that complex orthopaedic injuries may have different biological predictors to less severe orthopaedic injuries, which warrants further exploration and dedicated research.

#### Psychological factors

Beyond 6 months, Duong et al.,^11^ describe other psychological factors as predictors for RTW. This includes self-efficacy and recovery expectations. Conversely, this review only describes one study which tested and identified mental health as a predictor of RTW.^23^ This is in spite of the significant relationship between orthopaedic injury and depression, anxiety and post-traumatic stress disorder.^12,41^ This effect is so profound that up to 50% of orthopaedic patients suffer from mental health disorders.^42^ In response, early screening for PTSD/depression following orthopaedic injury is recommended to facilitate early mental health interventions.^43^ Therefore, future research into complex orthopaedic injury should include a mental health component to better explore how this impacts RTW in this patient population.

#### Social factors

Duong et al.,^11^ also describe social factors including blue-collar work (manual work), and physical workload as important predictors of RTW in orthopaedic patients. Similarly, this review describes physically demanding work as a prognostic factor in one paper^26^ but not another.^29^ No paper included in this review tested prognostic factors related to workplace adaptations such as job-modifications or a phased return to work.

Following injury or illness, patients commonly report barriers to RTW because of their employment.^44^ However, there are modifications reported in the literature that may help better address the social aspect of the biopsychosocial model. This could include a self-management programme to help the patient adapt to their workplace^45^ or the implementation of a sickness-absence model by the patient and the employer to promote work modification and a phased-RTW.^46^ Whilst the study of such modifications is likely more difficult than collecting data on the biological and psychological aspects of a patient's rehabilitation, future study on effective workplace modifications and support for complex orthopaedic patients is needed to facilitate their re-entry to employment.

### Future practice

The authors recommend that future studies into prognostic factors for RTW following complex orthopaedic injury encompass the following elements as a baseline between studies: (1) collect data on injury type (open fractures, multiple fractures, polytrauma, high energy pelvic fractures), ISS score, surgical complications, age, type of rehabilitation, type of work (manual vs non-manual), education status and compensation status; (2) complete the European Quality of Life questionnaire (EQ-5D-5L)^47^ to cover mobility, self-care, usual activities, pain or discomfort and anxiety or depression during recovery; (3) complete the Short Musculoskeletal Function Assessment (SMFA) questionnaire^48^ to contextualise their injuries and the bother this causes them. Together, these recorded variables encompass the biopsychosocial aspects of a patient's rehabilitation.

In addition, the authors also advocate the introduction of qualitative study into prognostic factors for RTW following complex orthopaedic injury. There are no such studies on this topic. Qualitative research is important as thematic analyses could offer novel insight into the patient experience of injury and recovery, and may identify new areas to target interventions.

### Limitations

As a scoping review, this paper does have its limitations. A risk of bias assessment was not conducted on collected papers, nor were original research contributions provided. However, this paper does highlight the striking lack of academically robust literature around prognostic factors for RTW following complex orthopaedic injury and has included study recommendations for future work on this topic.

## Conclusion

This scoping review was conducted to identify current literature, present recent evidence, and highlight gaps in knowledge of RTW following complex orthopaedic injury. This review has summarised the characteristics of 11 articles that explored RTW following complex orthopaedic injury. In Summary: (1) the majority of studies focused on open fractures of the lower leg; (2) no studies focused on open fractures of the upper limb; (3) there were two or fewer studies focused on multiple fractures, pelvic fractures and polytrauma; (4) there is no standardised follow up period; (5) there is limited consensus on prognostic factors. Overall, research into RTW following complex orthopaedic injury is incomplete and inconsistent despite poor patient outcomes. Therefore, there is a substantial opportunity for dedicated research on the prognostic factors of successful RTW in this patient subgroup, which should include qualitative investigation.

## References

[1] Silvester L Trompeter A Hing C . Patient experiences of rehabilitation following traumatic complex musculoskeletal injury – A mixed methods pilot study. Trauma 2022; 24: 218–225.

[2] Herron J Hutchinson R Lecky F , et al. The impact of age on major orthopaedic trauma: an analysis of the United Kingdom trauma audit research network database. Bone Joint J 2017; 99-B: 1677–1680. 29212692 10.1302/0301-620X.99B12.BJJ-2016-1140.R2

[3] National Audit Office. Major trauma care in England 2010, https://www.nao.org.uk/wp-content/uploads/2010/02/0910213.pdf .

[4] Cole E . The national major trauma system within the United Kingdom: inclusive regionalized networks of care. Emerg Crit Care Med 2022; 2: 76–79.

[5] National Institute for Health and Care Excellence (NICE). Rehabilitation after traumatic injury 2022, https://www.nice.org.uk/guidance/ng211 . 35471781

[6] Engel GL . The need for a new medical model: a challenge for biomedicine. Science 1977; 196: 129–136. 847460 10.1126/science.847460

[7] Severinsson Y Grimby-Ekman A Nordeman L , et al. Components of primary care multimodal rehabilitation and their association with changes in sick leave: an observational study. Work 2023; 74: 907–917. 36404565 10.3233/WOR-210836

[8] Pahlplatz TMJ Schafroth MU Krijger C , et al. Beneficial and limiting factors in return to work after primary total knee replacement: patients’ perspective. Work 2021; 69: 895–902. 34180460 10.3233/WOR-213522 PMC8385499

[9] Stewart D Al Hail M Al-Shaibi S , et al. A scoping review of theories used to investigate clinician adherence to clinical practice guidelines. Int J Clin Pharm 2023; 45: 52–63. 36385205 10.1007/s11096-022-01490-9 PMC9938823

[10] Amundsen O Moger TA Holte JH , et al. Patient characteristics and healthcare use for high-cost patients with musculoskeletal disorders in Norway: a cohort study. BMC Health Services Research 2024; 24: 1583. 39695620 10.1186/s12913-024-12051-3 PMC11653887

[11] Duong HP Garcia A Hilfiker R , et al. Systematic review of biopsychosocial prognostic factors for return to work after acute orthopedic trauma: a 2020 update. Front Rehabil Sci 2021; 2: 791351. 36188871 10.3389/fresc.2021.791351 PMC9397710

[12] Kalski L Claußen L Hofmann MA , et al. Health-related risk factors for subsequent work disability: a systematic literature review. Work 2025; 0: 10519815241290109. 10.1177/10519815241290109 39973649

[13] Clay FJ Newstead SV McClure RJ . A systematic review of early prognostic factors for return to work following acute orthopaedic trauma. Injury 2010; 41: 787–803. 20435304 10.1016/j.injury.2010.04.005

[14] McPeake J Mikkelsen ME Quasim T , et al. Return to employment after critical illness and its association with psychosocial outcomes. A systematic review and meta-analysis. Ann Am Thorac Soc 2019; 16: 1304–1311. 31184500 10.1513/AnnalsATS.201903-248OC

[15] Wagman P Gunnarsson AB Hjärthag F , et al. Quality of life, sense of coherence and occupational balance one year after an occupational therapy intervention for people with depression and anxiety disorders. Work 2023; 76: 561–568. 37066954 10.3233/WOR-220096 PMC10657701

[16] Figueredo JM Garcia-Ael C Gragnano A , et al. Well-being at work after return to work (RTW): a systematic review. Int J Environ Res Public Health 2020; 17: 7490. 33076302 10.3390/ijerph17207490 PMC7602369

[17] Hoogervorst LA Hart MJ Simpson PM , et al. Outcomes of severe lower limb injury with mangled extremity severity score ≥ 7. Bone Jt J 2021; 103: 769–774. 10.1302/0301-620X.103B4.BJJ-2020-1647.R1 33789468

[18] McMinn KR Thomas EV Martin KR , et al. Psychological morbidity and functional impairment following traumatic pelvic injury. Injury 2020; 51: 978–983. 32081393 10.1016/j.injury.2020.02.038

[19] Peters MDJ Godfrey C McInerney P , et al. Best practice guidance and reporting items for the development of scoping review protocols. JBI Evid Synth 2022; 20: 953–968. 35102103 10.11124/JBIES-21-00242

[20] Peters MDJ Marnie C Tricco AC , et al. Updated methodological guidance for the conduct of scoping reviews. JBI Evid Synth 2020; 18: 2119–2126. 33038124 10.11124/JBIES-20-00167

[21] Ouzzani M Hammady H Fedorowicz Z , et al. Rayyan-a web and mobile app for systematic reviews. Syst Rev 2016; 5: 210. 27919275 10.1186/s13643-016-0384-4 PMC5139140

[22] National Centre for Science and Engineering Statistics. Publciations Output: U.S. Trends and International Comparisons 2023 [cited 2024 15 January], https://ncses.nsf.gov/pubs/nsb202333/publication-output-by-region-country-or-economy-and-by-scientific-field .

[23] Belangero WD Fogagnolo F Kojima KE , et al. Isolated open tibial shaft fracture: a seven-hospital, prospective observational study in two Latin America countries. Rev Col Bras Cir 2022; 49: e20223301. 10.1590/0100-6991e-20223301-en PMC10578785 36449940

[24] Fioravanti M Maman P Curvale G , et al. Amputation versus conservative treatment in severe open lower-limb fracture: a functional and quality-of-life study. Orthop Traumatol Surg Res 2018; 104: 277–281. 29407071 10.1016/j.otsr.2017.12.013

[25] Frisvoll C Clarke-Jenssen J Madsen JE , et al. Long-term outcomes after high-energy open tibial fractures: is a salvaged limb superior to prosthesis in terms of physical function and quality of life? Eur J Orthop Surg Traumatol 2019; 29: 899–906. 30756177 10.1007/s00590-019-02382-x

[26] Levy JF Reider L Scharfstein DO , et al. The 1-year economic impact of work productivity loss following severe lower extremity trauma. J Bone Jt Surg 2022; 104: 586–593. 10.2106/JBJS.21.00632 35089905

[27] Major Extremity Trauma Research Consortium (METRC). Outcomes following severe distal tibial, ankle, and/or mid/hindfoot trauma: comparison of limb salvage and transtibial amputation (OUTLET). JBJS 2021; 103: 1588–1597. 10.2106/JBJS.20.01320 33979309

[28] Sethuraman AS Devendra A Rajasekaran RB , et al. Is lower limb salvage worthwhile after severe open tibial fractures in a developing country? An analysis of surgical outcomes, quality of life and cost implications. Injury 2021; 52: 996–1001. 33423773 10.1016/j.injury.2020.12.027

[29] Aprato A Joeris A Tosto F , et al. Are work return and leaves of absence after acetabular fractures predictable? : a retrospective study of 108 patients. Musculoskelet Surg 2017; 101: 31–35. 27734206 10.1007/s12306-016-0430-3

[30] Gabbe BJ Hofstee DJ Esser M , et al. Functional and return to work outcomes following major trauma involving severe pelvic ring fracture. ANZ J Surg 2015; 85: 749–754. 24889491 10.1111/ans.12700

[31] Smith JJ Agel J Swiontkowski MF , et al. Functional outcome of bilateral limb threatening: lower extremity injuries at two years postinjury. J Orthop Trauma 2005; 19: 249–253. 15795573 10.1097/01.bot.0000151813.10046.e4

[32] Kuhlman MB Lohse N Sørensen AM , et al. Impact of the severity of trauma on early retirement. Injury 2014; 45: 618–623. 24176678 10.1016/j.injury.2013.09.007

[33] Larsen P Goethgen CB Rasmussen S , et al. One-year development of QOL following orthopaedic polytrauma: a prospective observational cohort study of 53 patients. Arch Orthop Trauma Surg 2016; 136: 1539–1546. 27501705 10.1007/s00402-016-2550-5

[34] Hadfield JN Omogbehin TS Brookes C , et al. The open-fracture patient evaluation nationwide (OPEN) study. Bone & Joint Open 2022; 3: 746–752. 36181319 10.1302/2633-1462.310.BJO-2022-0079.R1 PMC9626856

[35] Schade AT Khatri C Nwankwo H , et al. The economic burden of open tibia fractures: a systematic review. Injury 2021; 52: 1251–1259. 33691946 10.1016/j.injury.2021.02.022

[36] Levy ML . The national review of asthma deaths: what did we learn and what needs to change? Breathe 2015; 11: 14–24. 26306100 10.1183/20734735.008914 PMC4487386

[37] Cech A Rieussec C Kerschbaumer G , et al. Complications and outcomes in 69 consecutive patients with floating hip. Orthop Traumatol: Surg Res 2021; 107: 102998. 34214653 10.1016/j.otsr.2021.102998

[38] Pallister I Handley GJ Maggs S , et al. Measuring recovery after open lower limb fractures: combined objective functional tests and global perceived recovery outperform narrower metrics and a standard generic patient reported outcome measure. BMC Musculoskelet Disord 2021; 22: 539. 34118896 10.1186/s12891-021-04356-9 PMC8199836

[39] Monteleone AS Feltri P Molina MN , et al. Quality of life from return to work and sports activities to sexual dysfunction after surgical treatment of acetabular fractures. Arch Orthop Trauma Surg 2023; 143: 1491–1497. 35218369 10.1007/s00402-022-04394-5

[40] Lindahl M Juneja H . I'll be back – predictive validity of adults’ expectations for recovery after fractures - A longitudinal observational study. Injury 2023; 54: 1553–1562. 10.1016/j.injury.2023.03.006 36925373

[41] Chen K Hynes KK Dirschl D , et al. Depression, anxiety, and post-traumatic stress disorder following upper versus lower extremity fractures. Injury 2024; 55: 111242. 38044162 10.1016/j.injury.2023.111242

[42] Scott S Brameier DT Tryggedsson I , et al. Prevalence, resources, provider insights, and outcomes: a review of patient mental health in orthopaedic trauma. J Orthop Surg Res 2024; 19: 538. 39223649 10.1186/s13018-024-04932-4 PMC11370264

[43] Weinerman J Vazquez A Schurhoff N , et al. The impacts of anxiety and depression on outcomes in orthopaedic trauma surgery: a narrative review. Annal Med Surg 2023; 85: 5523–5527. 10.1097/MS9.0000000000001307 PMC10619579 37920654

[44] Starik T Huber M Zeilig G , et al. Employment barriers questionnaire: development and determination of its reliability and validity. Work 2024; 79: 1255–1267. 38820058 10.3233/WOR-230736 PMC11612956

[45] Longtin C Tousignant-Laflamme Y Coutu MF . A logic model for a self-management program designed to help workers with persistent and disabling low back pain stay at work. Work 2020; 67: 395–406. 33044220 10.3233/WOR-203289

[46] Demou E Brown J Sanati K , et al. A novel approach to early sickness absence management: the EASY (early access to support for you) way. Work 2015; 53: 597–608. 26409380 10.3233/WOR-152137 PMC4927878

[47] EUROQOL. EQ-5D-5L 2024, https://euroqol.org/information-and-support/euroqol-instruments/eq-5d-5l/.

[48] Williams N . The short musculoskeletal function assessment (SMFA) questionnaire. Occup Med 2016; 66: 757. 10.1093/occmed/kqw140 27994082

